# Maintenance Chemotherapy With Chinese Herb Medicine Formulas vs. With Placebo in Patients With Advanced Non-small Cell Lung Cancer After First-Line Chemotherapy: A Multicenter, Randomized, Double-Blind Trial

**DOI:** 10.3389/fphar.2018.01233

**Published:** 2018-11-06

**Authors:** Qin Wang, Lijing Jiao, Shengfei Wang, Peiqi Chen, Ling Bi, Di Zhou, Jialin Yao, Jiaqi Li, Zhiwei Chen, Yingjie Jia, Ziwen Zhang, Weisheng Shen, Weirong Zhu, Jianfang Xu, Yong Gao, Yabin Gong, Ling Xu

**Affiliations:** ^1^Department of Oncology, Yueyang Hospital of Integrated Traditional Chinese and Western Medicine, Shanghai University of Traditional Chinese Medicine, Shanghai, China; ^2^Institute of Clinical Immunology, Yueyang Hospital of Integrated Traditional Chinese and Western Medicine, Shanghai University of Traditional Chinese Medicine, Shanghai, China; ^3^Department of Thoracic Surgery, Fudan University Shanghai Cancer Center, Shanghai, China; ^4^Lung Tumor Clinical Medical Center, Shanghai Chest Hospital, Shanghai Jiaotong University, Shanghai, China; ^5^Department of Oncology, First Hospital Affiliated to Tianjin College of Traditional Chinese Medicine, Tianjin, China; ^6^Department of Oncology, Changshu the 2nd People's Hospital, Jiangsu, China; ^7^Department of Oncology, Affiliated Jiang-yin Hospital of the Southeast University Medical College, Jiangsu, China; ^8^Department of Traditional Chinese Medicine, Ruijin Hospital Affiliated to Shanghai Jiaotong University School of Medicine, Shanghai, China; ^9^Department of Oncology, Shanghai Pulmonary Hospital, Tongji University School of Medicine, Shanghai, China; ^10^Department of Oncology, Shanghai East Hospital, Tongji University School of Medicine, Shanghai, China; ^11^Tumor Institute of Traditional Chinese Medicine, Longhua Hospital, Shanghai University of Traditional Chinese Medicine, Shanghai, China

**Keywords:** NSCLC, maintenance chemotherapy, chinese herb medicine formulas, adverse events, quality of life

## Abstract

**Background:** Chinese Herb Medicine Formulas (CHMF) was reported to improve the quality of life (QoL) in advanced NSCLC patients. The present study was designed to investigate whether maintenance chemotherapy plus CHMF in patients would improve QoL and progression-free survival (PFS).

**Methods:** Seventy-one patients were enrolled from 8 medical centers in China, and were randomly assigned to a maintenance chemotherapy plus CHMF group (*n* = 35) or a maintenance chemotherapy plus placebo group (*n* = 36). The outcome measures included PFS, Karnofsky performance status (KPS) scores, QoL (assessed with the lung cancer symptom scale (LCSS) questionnaire), and adverse events (AEs).

**Results:** Patients in the CHMF group showed significant improvements in median PFS (HR = 0.55, 95% CI 0.28–0.88, *P* = 0.019), KPS scores (*P* = 0.047), fatigue (cycle [C] 3: *P* = 0.03), interference with daily activities (C3: *P* = 0.04) and dyspnea (C2: *P* = 0.03) compared with patients in the placebo group. Compared with the placebo group, the incidence of AEs decreased in the CHMF group, including loss of appetite (C2: *P* = 0.011, C4: *P* = 0.004) and dry mouth (C4: *P* = 0.011).

**Conclusion:** The essential finding of our study is that maintenance chemotherapy combined with CHMF may prolong PFS, relieve symptoms, improve QoL and alleviate the side effects.

## Introduction

Lung cancer is one of the most common causes of cancer-related death all over the world. More than 50% of patients with lung cancer present with advanced disease at diagnosis. Non-small cell lung cancer (NSCLC) accounts for nearly 85% of all lung cancers and is associated with an overall 5-year survival rate of 18% (Ferlay et al., 2015; Siegel et al., 2018).

In the past decade, much attention has been paid to identifying a number of oncogenic driver mutations in NSCLC, including constitutive activation mutations within the epidermal growth factor receptor (EGFR) (Jackman et al., 2006; Rosell et al., 2009) or anaplastic lymphoma kinase (ALK) rearrangement (Soda et al., 2007). However, data currently available suggest that only patients with oncogenic drivers benefit from targeted therapies, whereas in patients without these oncogenic drivers, the outcome with targeted agents such as erlotinib and crizotinib are similarly poor (Cui et al., 2011). For most of patients, palliative cytotoxic chemotherapy is the only treatment option.

Maintenance treatment (MT) has been investigated extensively in recent years. After 4–6 cycles of first-line standard platinum-based combination chemotherapy, patients with no progressive disease might have a choice to receive MT to prolong a favorable clinical condition (Gerber and Schiller, 2013). MT can be classified into two categories: continuation MT and switch MT. Continuation MT includes no platinum cytotoxic agents or molecular targeted therapies like VEGF and EGFR inhibitors. For NSCLC patients without oncogenic drivers, continuation MT refers to the use of at least one of the drugs that is given in the first-line regimen. NCCN version 2.2013 recommends MT with pemetrexed or docetaxel, gemcitabine or bevacizumab, or bevacizumab plus pemetrexed, which is widely accepted as a standard therapeutic option.

However, prolonged treatment with cytotoxic agents will result in cumulative toxicity, and the overall survival (OS) benefit remains modest (Gentzler and Patel, 2014). Symptom burden could be reduced in MT as the number of cycle increases, since the most commonly seen grade 3–4 AEs were fatigue (5%), neutropenia (3–4%), anemia (3–4%) (Ciuleanu et al., 2009; Paz-Ares et al., 2012, 2013). This may be partly the reason to discontinue subsequent treatment. Hence, in the decision-making about MT, performance status, persisting toxicity after first-line chemotherapy and patients' choices concerning treatment options must be taken into account (Reck et al., 2014). Apart from physician assessment, toxicity can be comprehensively evaluated with patient-reported outcome measures, such as quality of life (QoL) (Sztankay et al., 2017).

Chinese Herb Medicine Formulas (CHMF) has been widely used in the management of disease, maintenance of health, and prolongation of life expectancy in Asian countries for NSCLC patients (Xu et al., 2014; Zhou et al., 2016). CHMF is a key modality of traditional Chinese medicine (TCM) system, in which two or more medicinal herbs are combined in a complex herbal formulation (Cheng et al., 2017). For patients with metastatic NSCLC, platinum-based chemotherapy combined with TCM was reported to significantly decrease the incident of nausea and vomiting, improve ECOG from 69 to 90% and show a better QoL variables score calculated by FACT-L subscales (Guo et al., 2017; Liao et al., 2017). Moreover, our previous studies have shown that CHMF enhances the chemotherapeutic effect, improve QoL and reduce the adverse effects of platinum-contained chemotherapeutics on NSCLC patients (Jiao et al., 2017; Wang et al., 2017; Gong et al., 2018).

As one of the most common strategies used in combination with MT clinically, for patients who show no progression after first-line chemotherapy, including those with poor QoL, oral Chinese herbal medicine can be considered an effective and safe MT strategy with significant increase in OS and progression-free survival (PFS), as well as significant improvement in performance status (Wang et al., 2017). However, whether CHMF added to maintenance chemotherapy could result in better survival and tolerance benefits has not been made clear in well-controlled, randomized studies. For this reason, we assessed the progression-free survival, AEs and QoL after CHMF vs. placebo for patients undergoing maintenance chemotherapy with no progressive disease after first-line standard chemotherapy.

## Materials and methods

### Study design and participants

A randomized, double-blind, placebo controlled, multicenter trial was conducted at 8 medical centers in China, with a maintenance chemotherapy plus CHMF group and a maintenance chemotherapy plus placebo group. Participants were enrolled from medical centers with the following eligibility criteria: patients were pathologically or cytologically confirmed of stage IIIa-IV NSCLC (International Union Against Cancer, version 7); efficacy evaluation of the first-line chemotherapy is progression-free, including complete response (CR), partial response (PR) and stable disease (SD); patients were at the age of 18–75 years old; patients had a KPS score of ≥70; patients had an estimated life expectancy of at least 12 weeks; participants had no major organ dysfunction, and had plan for chemotherapy maintenance; and informed consent was obtained from the patients. Patients were not eligible if they had malignant tumors except for NSCLC 5 years prior to study entry, received targeted therapy or other anticancer treatment, or were allergic to chemotherapy drugs.

### Ethics

The whole project was funded by Special Scientific Research for Traditional Chinese Medicine (China). Study of maintenance therapy including chemotherapy, Tyrosine Kinase Inhibitor (TKI) combined with CHMF or CHMF only were conducted synchronously. As one of subprojects, this study protocol was approved by the institutional review boards (IRBs) of the participating medical centers, the independent National Ethics Committee (EC), the Chinese Medicinal Agency, and was registered under the identifier NCT02900742 at the Clinicaltrials.Gov website. After the expire date, patients were followed up by Longhua Hospital affiliated to Shanghai University of TCM until September 21, 2017 which still reviewed by IRB. All participants provided written informed consent before enrolment.

### Sample size

No valid data on survival was available as few studies were designed to evaluate the efficacy of TCM combined with maintenance chemotherapy. The sample size of this study was based on the validity of assumptions and clinical experience of the past studies (Pérol et al., 2012; Wang et al., 2013; Yifen et al., 2014). A two-sided log-rank test with an overall sample size of 114 subjects (57 in the control group and 57 in the treatment group) achieved 80% power at a 0.05 significance level when the median survival time was 6 and 3 months respectively in the treatment group and the control group. The study lasted for 36 months, with subject accrual (entry) occurring in the first time period. The drop-out rate was 10% in the control group.

### Randomization and blinding

Eligible patients were randomly assigned 1:1 to receive maintenance chemotherapy with CHMF or with placebo. Central randomization was performed by a clinical research organization (CRO) (Shanghai Clinical Research Center, Shanghai, China http://www.scrcnet.org). Random numbers were automatically generated by computer referring to pre-configured stratified factors (smoking status (smoker, ex-smoker or non-smoker), gender (female, male), age (>70, ≤ 70) and chemotherapeutics (pemetrexed, gemcitabine, and docetaxel) before administration of maintenance chemotherapy. Then the CRO provided the random number to each participating center. The block size and treatment-assignment table were not available to the investigators and patients until the end of the study.

### Intervention procedure

Patients received continuation maintenance chemotherapy (gemcitabine 1,250 mg/m^2^, ivgtt 30 min on days 1 and 8, once every 21 days or pemetrexed 500 mg/m^2^, ivgtt 30 min on day 1, once every 21 days or docetaxel 75 mg/m2, ivgtt 30 min on day 1, once every 21 days) until evidence of disease progression or unacceptable toxicity. Patients in maintenance chemotherapy+CHMF or maintenance chemotherapy+ placebo group received treatment with CHMF or matched placebo respectively. CHMF and placebo granules were administered on the first day after chemotherapy. The medicine was dissolved in 160 mL of warm water for drinking at 9:30 a.m. and 15:00 p.m. every day until the end of chemotherapy or unacceptable toxicity. All procedures were performed under the supervision of clinical research pharmacists. The processed herbs and the granules were prepared in compliance with Good Manufacturing Practice (GMP).

### CHMF preparation and chemical structure analysis by HPLC

**Figure 1:**
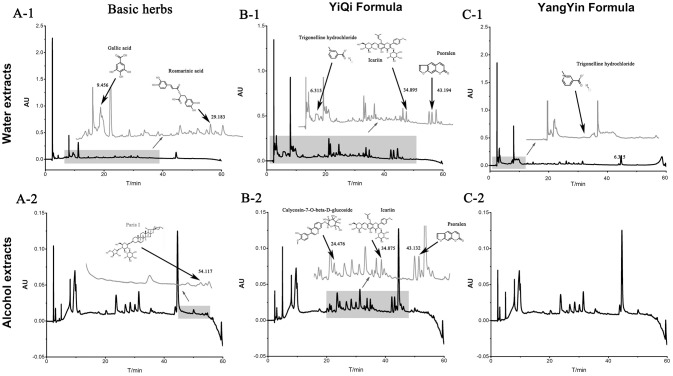
The water and alcohol extracts of three formulations were analyzed by high performance liquid chromatography (HPLC) in 203 nm.

Three formulas (batch number: 1208304, 1208303, and 1207357) were dynamic cycle extracted, then concentrated, spray-dried to make granules by Jiangyin Tian Jiang Pharmaceutical Co. Ltd. (Jiangsu, China). Besides, free of bacteria, mold and Escherichia coli were also monitored by this company before ready to use. The water and alcohol extracts of three formulations were analyzed by high performance liquid chromatography (HPLC) for quality control. Methanol from Yuwang (China) was used for HPLC analysis and LC-MS. Acetonitrile of HPLC grade from J&K (Beijing, China) was used for purity analysis by UPLC. Preparative grade n-hexane, isopropanol, ethyl acetate and methanol were purchased from Shilian (Anhui, China). All preparative reagents were filtered through a 0.22 mm organic membrane. The water used in this study was purified with a Milli-Q water purification system (Millipore, Bedford, MA, USA). Purity analysis of compounds was performed on a Waters ACQUITY UPLC® H-Class system with a PDA detector. The standards (1.00 mg) were prepared with 1 mL methanol, then filter by Nylon membrane filter (0.22 μm). The solution was further analysis by HPLC. As shown in Figure 1, the bioactive components were extracted including Rosmarinic acid (C18H16O8; CAS: 20283-92-5), Gallic acid (C7H6O5; CAS: 149-91-7) and Paris I (C44H70O16; CAS: 50773-41-6) in basic formula, Icariin (C33H40O15; CAS: 489-32-7) Psoralen (C11H6O3; CAS: 66-97-7) Calycosin-7-O-beta-D-glucoside (C22H22O10; CAS: 20633-67-4) and Trigonelline hydrochloride (C7H8ClNO2; CAS: 6138-41-6) in YiQi formula, and Trigonelline hydrochloride (C7H8ClNO2; CAS: 6138-41-6) in YangYin formula.

### Placebo

In conformity with Good Clinical Practice (GCP), in double-blind clinical trials, the investigational drug and placebo should be identical in appearance (color, viscosity, hardness, and other physical properties), smell, packaging, labeling, and other characteristics (Xu et al., 2012; Jiao et al., 2017). It is difficult to define a placebo in Chinese herbal medicine where all-natural substances are potentially therapeutic. Therefore, only food color and artificial flavors included in the raw materials for the placebo. Ingredient including:

Yangyin formula (placebo): Lactose (94.06%): citric yellow pigment (0.15%): sunset yellow pigment (0.09%): caramel pigment (4.1%): citric acid (0.8%): bitterness (0.8%)

Yiqi formula (placebo): Lactose (94.96%): citric yellow pigment (0.2%): sunset yellow pigment (0.04%): caramel pigment (3.3%): bitterness (1.5%)

Basic herbs (placebo): Lactose (92.37%): citric yellow pigment (0.1%): sunset yellow pigment (0.03%): caramel pigment (6.3%): bitterness (1.2%).

### Syndrome differentiation criteria

The CHMFs were prescribed based on syndrome differentiation. Syndrome differentiation criteria were based on “The Guiding Principles of Clinical Research of New Chinese Medicine (trial)” (China Pharmaceutical Technology Publishing House, 2002) and “Shanghai diagnosis and treatment routine of TCM Syndrome”(Shanghai Municipal Health Bureau edit). Three syndromes of TCM are set as follows: Qi deficiency syndrome is composed of the following symptoms: cough with phlegm, poor appetite, lassitude and weakness, pale and plump tongue; secondary symptoms of spontaneous sweating, loose stool or soft slippery pulse.

Yin deficiency syndrome contains main symptoms of cough with scanty phlegm, dry mouth, reddish tongue; secondary symptoms of night sweating, heartburn and insomnia, low fever, thread and rapid pulse.

Qi and Yin deficiency syndrome contains main symptoms of cough with scanty phlegm, shortness of breath, lassitude and weakness, thirst without the desire to drink; secondary symptoms of spontaneous sweating, night sweating, reddish tongue or tongue with teeth marks, thread and weak pulse. The diagnosis can be given with at least two of the main symptoms and one of the secondary symptoms. Each formula was prescribed by one professor who had worked in our hospital for more than 50 years to facilitate clinical research. Patients who diagnosed with Qi syndrome deficiency, Yin syndrome deficiency received treatment with basic herbs adding YiQi formula and YangYin formula respectively. Patients with both Qi and Yin syndrome deficiency prescribed with basic herbs adding the combination of YiQi and YangYin formula granules. In placebo group, patients have the corresponding placebo granules. The composition of the formulas was shown in Table 1.

**Table 1:** Composition of the basic herbs and added formulas with different syndromes.

**Chinese name**	**Chinese name (Pinyin)**	**Pharmaceutical name**	**English name**	**Dosage (grams)**
**BASIC HERBS FOR ALL PATIENTS**				
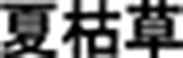	Xiakucao	*Prunlla vulgaris* L.	Spica Prunellae	7.5
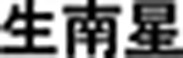	Shengnanxing	*Arisaema erubescens* (Wall.) Schott.	Arisaema Rhizoma Arisaematis	15
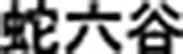	Sheliugu	*Amorphophallus konjac* K.Koch	Rhizoma Amorphophalli	15
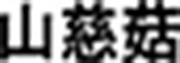	Shancigu	*Cremastra appendiculata* (D.Don) Makino	Pseudobulbus Cremastrae Seu Pleiones	7.5
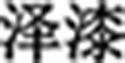	Zeqi	*Euphorbia helioscopia* L.	Euphorbiae Helioscopiae	7.5
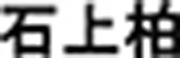	Shishangbai	*Selaginella Doederleinii* Hieron	Selaginella Doederleinii	15
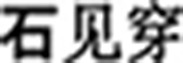	Shijianchuan	*Salvia chinensis* Benth.	Salviae Chinensis	15
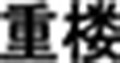	Chonglou	*Paris polyphylla* Smith var. chinensis (Franch.) H.Hara	Rhizoma Paridis	7.5
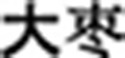	Dazao	*Ziziphus jujuba* Mill.	Fructus Jujubae	4.5
**FOR QI SYNDROME DEFICIENCY, ADD YIQI FORMULA**				
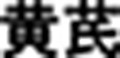	Huangqi	*Astragalus membranaceus* Fisch. ex Bunge	Milkvetch Root Radix Astragali	30
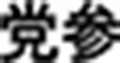	Dangshen	*Codonopsis pilosula* (Franch.) Nannf.	Codonopsis Radix	9
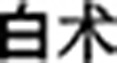	Baizhu	*Atractylodes macrocephala* Koidz.	Atractylodis Macrocephalae Rhizoma	12
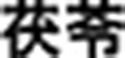	Fuling	*Poria cocos* (Schw.) Wolf.	Indian Bread Poria	15
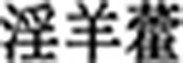	Yinyanghuo	*Epimedium brevicornu* Maxim.	Epimedii Folium	15
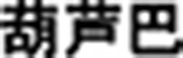	Huluba	*Trigonella foenum-graecum* L.	Common Fenugreek Seed Semen Trigonellae	15
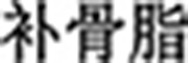	Buguzhi	*Cullen corylifolium (L.)* Medik.	Fructus Psoraleae	12
**FOR YIN SYNDROME DEFICIENCY, ADD YANGYIN FORMULA**				
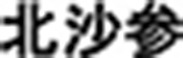	Beishashen	*Glehnia littoralis* (A.Gray) F.Schmidt ex Miq.	Coastal Glehnia Root	30
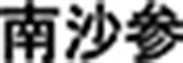	Nanshashen	*Adenophora stricta* Miq.	Fourleaf Ladybell Root	30
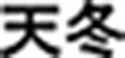	Tiandong	*Asparagus cochinchinensis* (Lour.) Merr.	Cochinchinese Asparagus Root	15
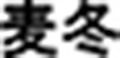	Maidong	*Ophiopogon japonicus* (Thunb.) Ker Gawl.	Dwarf Lilyturf Tuber	15
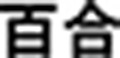	Baihe	*Lilium brownii* var. viridulum Baker	Lilii Bulbus	15
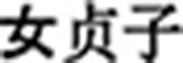	Nvzhenzi	*Ligustrum lucidum* W.T.Aiton	Fructus Ligustri Lucidi	12

### Follow-up

Follow-up was carried out at 2, 4, and 6 months to assess the clinical response and tolerance. Tumor imaging assessments (MRI or CT) were performed at screening, at the end of C2, and then once every two cycles until the end-of-treatment visit. Objective tumor response was measured using RECIST version 1.1 and assessed by the investigator and by a blinded independent review committee (IRC) and Blood chemistry, hematology, concomitant medications, and adverse events were assessed on day 1 of each 21-day cycle. Patient-reported outcomes were assessed at day 1 (baseline), and every 2 cycles, on day 1 of the subsequent cycles, at the end-of-treatment visit, and at the post-treatment follow-up visit, using the lung cancer symptom scale (LCSS). Patients who had progressive disease continue to be assessed every 3 months.

### Outcomes and adverse events

The primary endpoint of the study was progression-free survival (PFS), defined as the time interval between the date of enrolment and the date of disease progression or death by any cause. The secondary endpoints were LCSS, Karnofsky Performance Status (KPS) score and AEs of the regimen. Adverse events including toxicity and side effects were reported according to Common Terminology Criteria for Adverse Events V3.0 (CTC AE) issued by National Cancer Institute (NCI) (https://ctep.cancer.gov/protocolDevelopment/electronic_applications/docs/ctcaev3.pdf). All of the unexpected responses possibly or definitely related to the research were reported. In case of serious adverse events (SAEs), experimental treatment was stopped immediately and appropriate treatment was provided. The type and frequency of adverse events were reported for each group.

### Statistical analysis

Statistical analysis was performed using the SPSS software (version 18). For variable data (baseline), a chi-square test was used. Descriptive statistics were used to summarize patient characteristics by treatment group. Patients that had received at least one of the intended therapies were included in efficacy analysis. PFS was compared between the maintenance chemotherapy plus CHMF group and the maintenance chemotherapy plus placebo group using the log-rank test. Analysis of PFS was performed in all patients who had at least one post-baseline imaging assessment (modified intention-to-treat population). The KPS score “improvement and stability rates” after treatment, which was analyzed using a chi-square test, was categorized into improved (decrease in score ≥ 10), stable (score not changed) or worse (increase in score ≥ 10). Ordered hierarchical data (NCI-CTC graded AEs) were analyzed using a rank sum test. LCSS analysis was conducted on the QoL population, defined as the population of patients with a completed LCSS assessment (one question from the LCSS) at baseline and at least once during the study. The time to worsening of symptoms (TWS) was measured from the date of randomization to the date of a first clinically meaningful worsening for each of the LCSS items. Worsening of symptoms was defined as a change of one-half standard deviation, determined from the scores of corresponding baseline items (Boye et al., 2016). TWS was censored at the date of the last LCSS assessment for patients with unknown LCSS status or lost to follow-up and was analyzed using the Kaplan-Meier estimator. HRs were estimated by unadjusted Cox regression analysis, with assigned treatment as the only covariate. The mean maximum improvement from the baseline score was calculated for each of the LCSS items. Results were considered statistically significant if *P* ≤ 0.05.

## Results

### Participant characteristics

**Figure 2:**
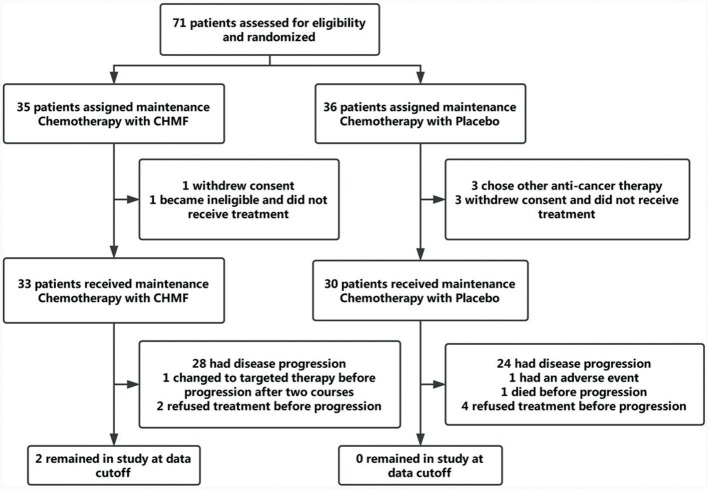
Study profile. Data cutoff was September 21, 2017. CHMF, Chinese herbal medicine formula.

Between July 22, 2013, and April 7, 2016, 71 patients were finally enrolled. As TKIs and CHMF alone as maintenance therapy after first line treatment were popular, it was hard to enroll patients who chose to receive chemotherapy. In April 2016, the interim analysis was conducted by the Data and Safety Monitoring Board which composed of biostatisticians from a clinical research organization (CRO) and clinicians. The result showed a significance improvement of PFS in maintenance chemotherapy plus CHMF compared with maintenance chemotherapy plus placebo [HR (95%) = 0.50 (0.28–0.92), *P* = 0.025]. So, the enrollment was ended before reaching the target number. The Data and Safety Monitoring Board kept the results confidential, and follow-up was conducted by our investigators until September 21, 2017. Of 71 patients, 35 were assigned to receive maintenance chemotherapy with CHMF and 36 to receive maintenance chemotherapy with placebo. During face-to-face interviews, the study protocol and objective were explained to patients in the hospital. Three patients from the placebo group and one from the CHMF group refused chemotherapy immediately after randomization. Three patients in the placebo group withdrew for personal reasons. One patient in the CHMF group became ineligible for the study. These patients were excluded from analysis. At the time of data cutoff (September 21, 2017), 6% (2/33) of patients in the CHMF group but no patients in the placebo group were still receiving treatment. Sixty-one patients discontinued the study, including 31 assigned to receive maintenance chemotherapies plus CHMF and 30 assigned to receive maintenance chemotherapy plus placebo (Figure 2). The most common reason for study discontinuation was disease progression, occurring in 84.8% (28/33) of patients who were assigned to receive maintenance chemotherapy plus CHMF and in 80% (24/30) of patients who were assigned to receive maintenance chemotherapy plus placebo. In the CHMF population lost to follow-up, 1 patient switched to targeted therapy after two treatment cycles and 2 patients refused treatment before progression. In the placebo population lost to follow-up, 1 patient have a serious adverse event after one treatment cycle, 4 patients refused treatment before progression, and 1 patient died before progression. These patients were included in the intention-to-treat efficacy analysis as shown in Figure 2. Baseline characteristics and stratification factors were well balanced in the two groups as shown in Table 2.

**Table 2:** Baseline characteristics and demographics of the population received maintenance treatment.

	**Maintenance Chemo + CHMF**	**Maintenance Chemo + Placebo**	***P*-value**
	**(*n* = 33)**	**(*n* = 30)**
**Gender, n (%)**			0.777
Male	22(66.7%)	21(70.0%)
Female	11(33.3%)	9(30.0%)
**Age, n (%)**	58.8 ± 8.9	58.0 ± 9.4	0.733
≥65	8(24.2%)	10(33.3%)	0.425
< 65	25(75.8%)	20(66.7%)
**KPS, n (%)**			0.702
90	24 (38.1%)	20 (31.7%)
80	6 (9.6%)	8 (12.7%)
70	3 (4.7%)	2 (3.2%)
**Smoking history, n (%)**			0.942
Smoked	19 (57.6%)	17 (56.7%)
Never smoke	14 (42.4%)	13 (43.3%)
**Pathological type, n (%)**			0.215
AC	29 (87.9%)	21 (70.0%)
SCC	3 (9.1%)	7 (23.3%)
Other NSCLC	1 (3.0%)	2 (6.7%)
**cTNM stage, n (%)**			0.183
IIIa	4 (12.1%)	3(10.0%)
IIIb	1 (3.0%)	5(16.7%)
IV	28 (84.8%)	22(73.3%)
**Chemo regimens, n (%)**			0.873
PEM	20(60.6%)	17(56.7%)
GEM	9(27.3%)	8(26.7%)
DOC	4(12.1%)	5(16.7%)
**TCM syndrome, n (%)**			0.617
Qi deficiency	10(30.3%)	12(40.0%)
Yin deficiency	4(12.1%)	2(6.7%)
Qi-Yin deficiency	19(57.6%)	16(53.3%)
**LCSS symptomsa, mean (range)**		
Loss of appetite	28.2 (0–80)	31.1 (0–70)	0.652
Fatigue	30.4 (0–80)	33.9 (0–70)	0.631
Cough	22.6 (0–80)	31.7 (0–70)	0.156
Dyspnea	16.7 (0–50)	22.2 (0–60)	0.29
Hemoptysis	7.4 (0–60)	11.1 (0–40)	0.368
Pain	13.0 (0–70)	17.8 (0–50)	0.33
Overall symptoms	30.4 (0–80)	37.2 (10–90)	0.324
Interference with daily activities	35.6 (0–90)	37.8 (0–90)	0.759
Overall QoL	27.8 (0–50)	35 (0–80)	0.227

*Data are expressed as numbers (percentage) or mean (range). Chemo, chemotherapy; CHMF, Chinese herbal medicine formula; KPS, Karnofsky performance status; PEM, pemetrexed; GEM, Gemcitabine; DOC, Docetaxel; LCSS, Lung Cancer Symptom Scale*.

**Figure 3:**
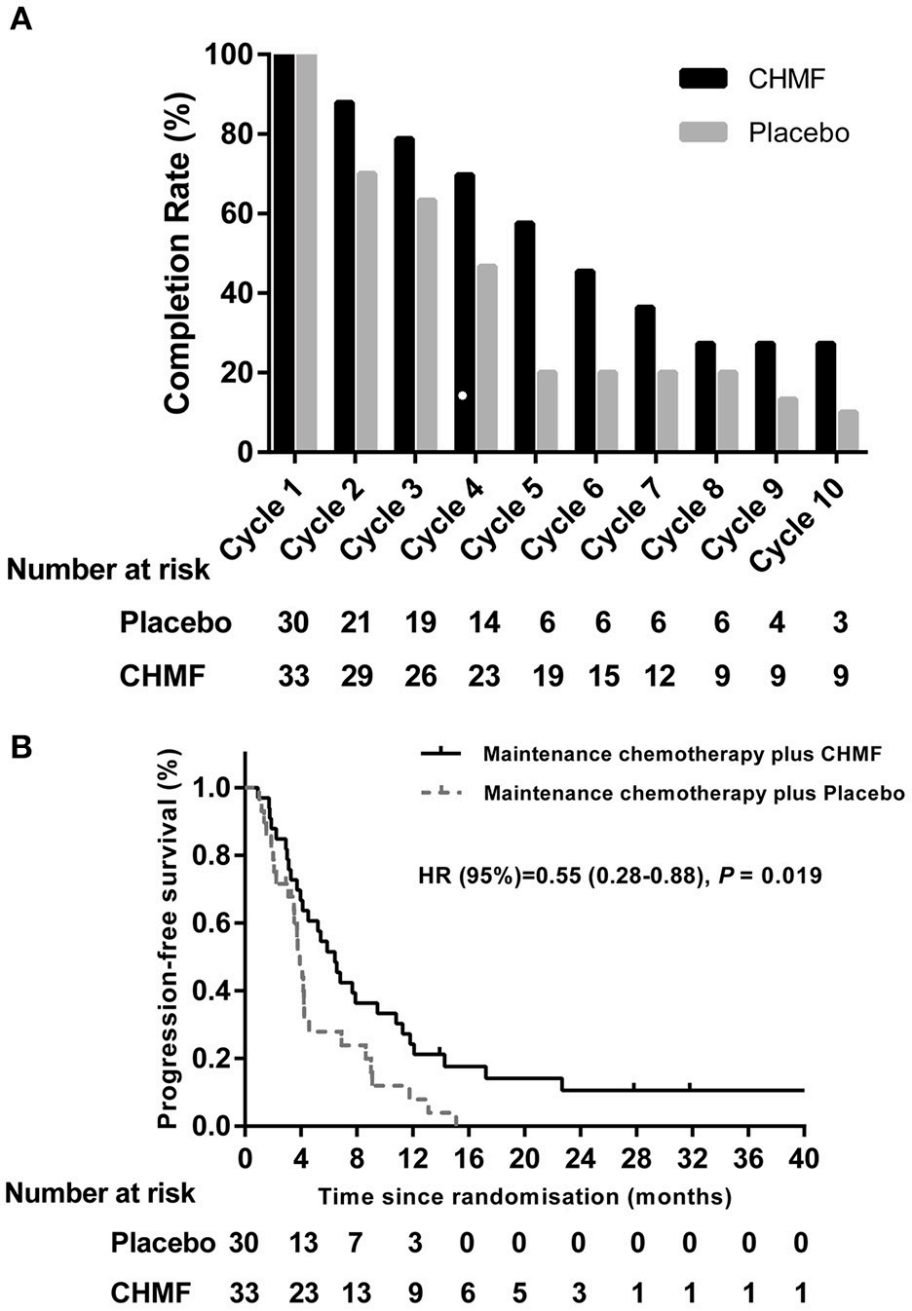
**(A)** Summary of maintenance cycle administered in the CHMF (Black) and placebo (Gray) arms of patients. CHMF, Chinese herbal medicine formula. **(B)** Progression-free survival. Kaplan-Meier estimates of progression-free survival as assessed by investigators in populations received maintenance treatment between CHMF (Black) and placebo (Gray) arms. Patients who had progression of tumor were censored on their last evaluable date. *P*-values were calculated using a two-sided log-rank test. HR, Hazard ratio; CI, confidence interval; CHMF, Chinese herbal medicine formula.

The treatment cycles were summarized in Figure 3A. In the overall population, 45.45% of patients in the CHMF group and 20.0% of patients in the placebo group remained on study treatment for at least 6 cycles. In addition, at the end of cycle 10, 27.21% of patients receiving maintenance chemotherapy with CHMF remained on study treatment, compared to 10.0% of those receiving maintenance chemotherapy with placebo. In the overall population, no patient in the two groups experienced dose delays due to AEs.

### Effects on PFS

After a median follow-up of 4.18 months (IQR 2.95~9.00), the 3-, 6-, and 12-month progression-free survival was 78.8 vs. 71.5%, 51.5 vs. 27.9%, and 21.2 vs. 8% in the CHMF and placebo groups. Median PFS (Figure 3B) from randomization was 6.43 months (3.27–11.80) in the CHMF group and 3.73 months (2.07–4.60) in the placebo group (HR 0.55, 95% CI 0.28–0.88, *P* = 0.019).

### Effects on the KPS score and symptoms

The KPS scores “improvement and stability rates” were 93.3% in the CHMF group vs. 70% in the placebo group (*P* = 0.047, 95% CI 0.03–0.935).

**Figure 4:**
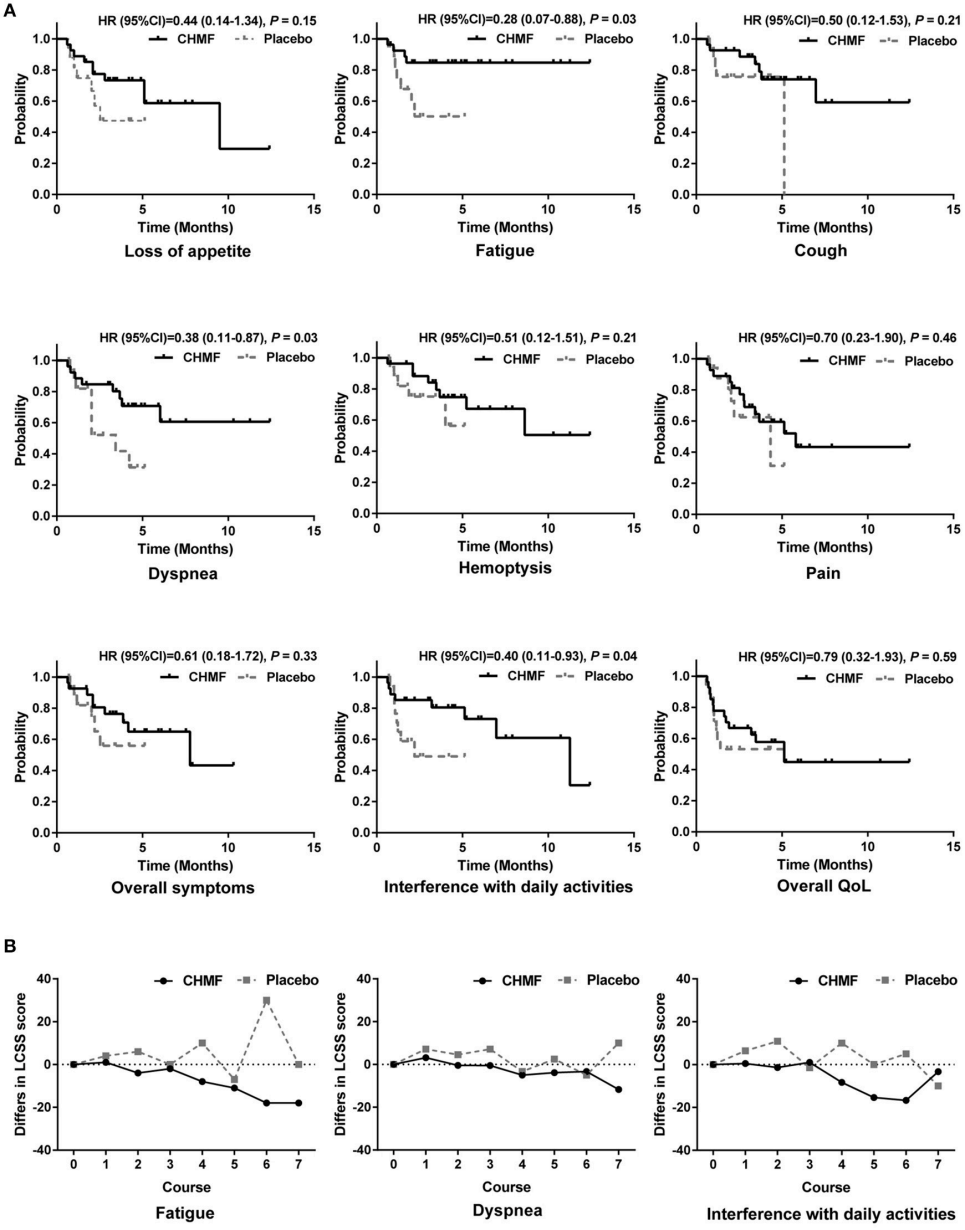
Quality of life in patients evaluated by LCSS analyses. **(A)** Comparison of time to worsening of symptoms between CHMF (Black) and placebo (Gray) arms. Probabilities were reported as Hazard Ratios (HRs) With 95% Confidence Intervals. **(B)** Comparison of changes from baseline in LCSS symptom score at each visit from cycle 1 to 7. Lower values represent lower symptom severity, less Interference with normal activities. CI, confidence interval; HR, hazard ratio; LCSS, Lung Cancer Symptom Scale; CHMF, Chinese herbal medicine formula.

In the QoL population as shown in Figure 4, more improvements were observed in the LCSS scores from baseline in the CHMF group than in the placebo group. In the CHMF group, after each of the 3 cycles of chemotherapy, median decrease in score from baseline was seen for fatigue (HR = 0.28, 0.07–0.88, *P* = 0.03) and interference with daily activities (HR = 0.40, 0.11–0.93, *P* = 0.04), indicating improvement. Improvement was also seen in the dyspnea score (HR = 0.38, 0.11–0.87, *P* = 0.03) after 2 cycles of chemotherapy. No change or worsening in the LCSS scores was seen for all of the other symptoms, including overall symptoms, pain, and overall QoL.

### Effects on AEs

**Figure 5:**
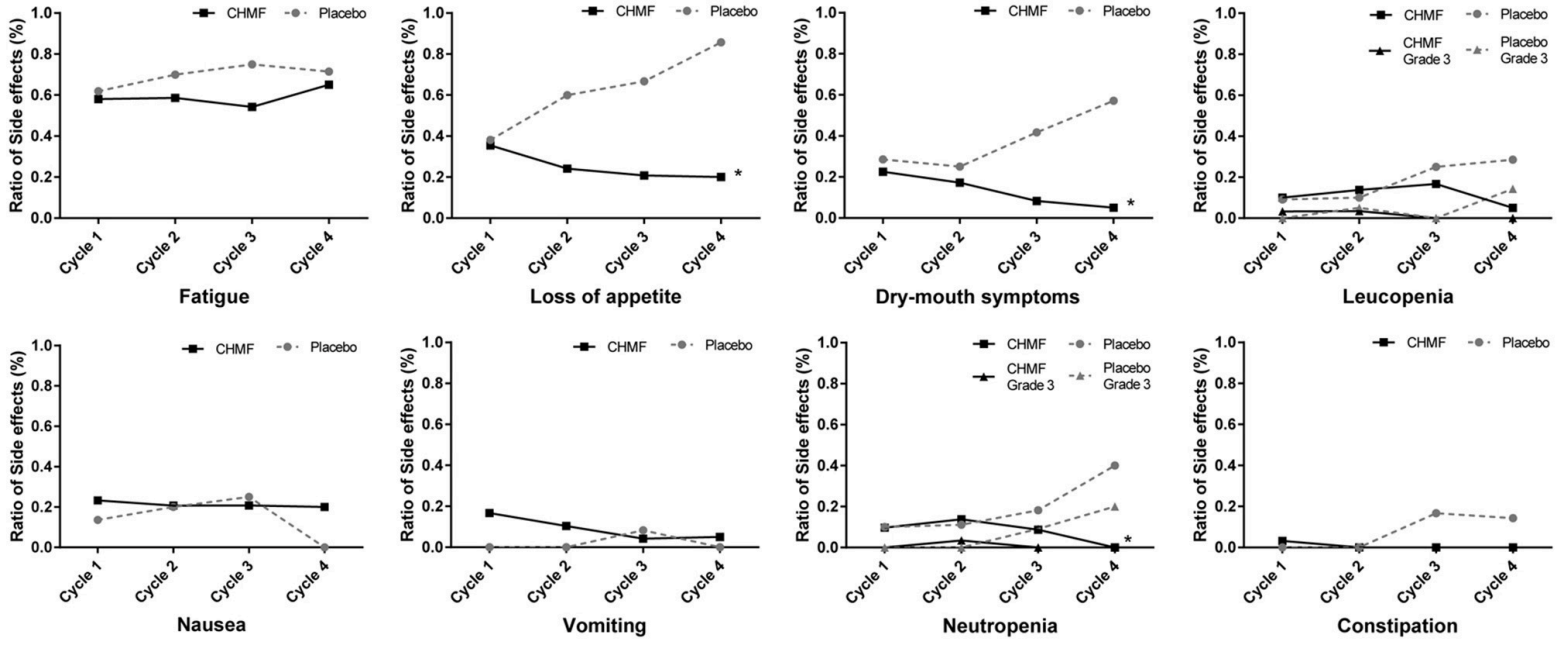
Figures present adverse events occurring in >15% of patients (Fatigue, Loss of appetite, Dry mouth, Leucopenia, Nausea, Vomiting, Neutropenia) and Constipation during first four maintenance Cycles between patients in the CHMF (Black) and placebo (Gray) arms. The dotted line stands for adverse event of Grade 3. *Stands for *P* < 0.05. CTCAE, Common Terminology Criteria for Adverse Events; CHMF, Chinese herbal medicine formula.

Among the 63 patients that received maintenance chemotherapy, one patient refused chemotherapy for drug nephrotic syndrome. The most common grade 1 or 2 toxicities occurring in >15% of patients (Table 3) were fatigue, loss of appetite, dry mouth, leucopenia, nausea, vomiting and neutropenia. After two cycles of treatment (Figure 5), the most common grade 1 or 2 toxicities were asthenia or fatigue (17 of 29 [58.7%] patients in the CHMF group vs. 14 of 20 [70%] patients in the placebo group, *P* = 0.42), loss of appetite (7 of 29 [24.1%] patients in the CHMF group vs. 12 of 20 [60%] patients in the placebo group, *P* = 0.011) and dry mouth (5 [17.2%] vs. 5 [25.0%]). One grade 3 serious adverse events (SAEs) were reported in the CHMF group and three in the placebo group. However, after four treatment cycles, the incidence of symptoms increased as follows: asthenia or fatigue (13 of 20 [65.0%] patients in the CHMF group vs5 of 7 [71.4%] patients in the placebo group, *P* = 0.76), loss of appetite (4 of 20 [20.0%] patients in the CHMF group vs. 6 of 7 [85.7%] patients in the placebo group, *P* = 0.004), and dry mouth (1 [5.0%] vs. 4 [57.1%], *P* = 0.011). However, three grade-3 SAEs were reported in the placebo group.

**Table 3:** Adverse events during the maintenance phases.

	**Maintenance chemo** + **CHMF (*****N*** = **31) n (%)**			**Maintenance chemo** + **Placebo (*****N*** = **22) n (%)**		
	**Grade 1/2**	**Grade 3**	**Any**	**Grade 1/2**	**Grade 3**	**Any**
Fatigue	28 (90.3%)	0	28 (90.3%)	22 (100%)	0	22 (100%)
Loss of appetite	15 (48.4%)	0	15 (48.4%)	16 (72.7%)	0	16 (72.7%)
Dry mouth	10 (32.3%)	0	10 (32.3%)	11 (50%)	0	11 (50%)
Leucopenia	8 (25.8%)	0	8 (25.8%)	2 (9.1%)	2 (9.1%)	4 (18.2%)
Nausea	8 (25.8%)	0	8 (25.8%)	6 (27.3%)	0	6 (27.3%)
Vomiting	5 (16.1%)	0	5 (16.1%)	1 (4.5%)	0	1 (4.5%)
Neutropenia	4 (12.9%)	1 (3.2%)	5 (16.1%)	2 (9.1%)	2 (9.1%)	4 (18.2%)
Elevated GGT	4 (12.9%)	0	4 (12.9%)	1 (4.5%)	1 (4.5%)	2 (9.1%)
Anemia	3 (9.7%)	0	3 (9.7%)	2 (9.1%)	1 (4.5%)	3 (13.6%)
ALT/AST increased	3 (9.7%)	0	3 (9.7%)	6 (27.3%)	0	6 (27.3%)
Arhythmia	3 (9.7%)	0	3 (9.7%)	0	1 (4.5%)	1 (4.5%)
Pain	3 (9.7%)	0	3 (9.7%)	4 (18.2%)	0	4 (18.2%)
Elevated cRE	2 (6.5%)	0	2 (6.5%)	0	0	0
Alopecia	2 (6.5%)	0	2 (6.5%)	1 (4.5%)	0	1 (4.5%)
Thrombocytopaenia	1 (3.2%)	0	1 (3.2%)	3 (13.6%)	0	3 (13.6%)
Elevated ALP	1 (3.2%)	0	1 (3.2%)	0	0	0
Elevated TB	1 (3.2%)	0	1 (3.2%)	0	0	0
Proteinuria	1 (3.2%)	0	1 (3.2%)	1 (4.5%)	0	1 (4.5%)
Weight-decrease	1 (3.2%)	0	1 (3.2%)	1 (4.5%)	0	1 (4.5%)
Constipation	1 (3.2%)	0	1 (3.2%)	2 (9.1%)	0	2 (9.1%)
Diarrhea	1 (3.2%)	0	1 (3.2%)	3 (13.6%)	0	3 (13.6%)
Pruritus	1 (3.2%)	0	1 (3.2%)	1 (4.5%)	0	1 (4.5%)
Esthesionosis	1 (3.2%)	0	1 (3.2%)	0	0	0
Rash	0	0	0	1 (4.5%)	0	1 (4.5%)
Haemoptysis	0	0	0	1 (4.5%)	0	1 (4.5%)

*Values are expressed as numbers (percentage). Table presents grade 1/2 and 3 adverse events in all patients during the maintenance phases. Adverse events are listed in descending order of frequency in the total patient population. ALT, alanine aminotransferase. AST, aspartate aminotransferase. GGT, γ-glutamyltransferase; CRE, serum creatinine; ALP, alkaline phosphatase; TB, total bilirubin*.

## Discussion

Maintenance chemotherapy refers to systemic therapy for patients with advanced NSCLC if the response is CR, PR, or SD after 4-6 cycles of first-line platinum-based chemotherapy (Gridelli et al., 2012). In China, TCM therapy is applied in the whole process of treatment, including conventional and subsequent therapy to maintain efficacy (Wang et al., 2017; Xiao et al., 2018). Clinically, if a patient with advanced NSCLC who has a performance status (PS) score of 0-2 and shows no disease progression after first line treatment, CHMF can be used in combination with single-agent chemotherapy. If PS of the patient is 3-4, CHMF will be administered to the patient alone as MT (Jiang et al., 2016). In both cases, CHMFs were used and had the effects of relieving symptoms, improving QoL, alleviating the side effects caused by chemotherapy, reducing relapse and metastasis, and enhancing short- and long-term therapeutic effects (Xu et al., 2014; Xiao et al., 2018). To the best of our knowledge, this is the first multicenter, randomized, and double-blind trial to demonstrate the advantages of single-agent maintenance chemotherapy combined with CHMF in improving PFS significantly.

In this study, efficacy and safety of maintenance chemotherapy used in combination with pemetrexed/gemcitabine/docetaxel and CHMF were evaluated in patients with advanced non-squamous NSCLC. The median PFS from randomization to the CHMF group in our study was 6.43 months (3.27–11.80), which was comparable with that reported in the Ciuleanu T study on pemetrexed maintenance therapy (4.3 months) and was longer than those reported in the Pérol M study on gemcitabine maintenance therapy (3.8 months) and the L Zhang study on docetaxel maintenance therapy (5.4 months) (Ciuleanu et al., 2009; Pérol et al., 2012; Zhang et al., 2013).

Toxicity after first-line chemotherapy was the main reason for refusing maintenance chemotherapy in patients with non-progressive disease. Maintenance chemotherapy could also be discontinued due to symptom burden such as fatigue, neutropenia, nausea and vomiting, constipation and appetite loss (Sztankay et al., 2017). Although MT with the three chemotherapeutics provides improved PFS and leads to similar grade 3/4 adverse events (Hu et al., 2016), it was reported that low-grade toxicities were potentially burdensome for patients. PFS benefits are regarded as favorable when symptoms are mild and as detrimental when symptoms are severe (Gridelli et al., 2012). In our study, there were one grade 3 serious adverse event reported in the CHMF group and 7 in the placebo group. In addition, 2 and 4 patients in the groups respectively discontinued MT before progression. The most common grade 1 or 2 toxicities occurring in >15% of patients were fatigue, loss of appetite, dry mouse, leucopenia, nausea, vomiting and neutropenia. This is consistent with other treatment preference studies, where severity of treatment-related side effects played a key role in patients' attitudes toward continued treatment (Brodowicz et al., 2006; Cella and Patel, 2008). In the maintenance chemotherapy + CHMF group, the incidence of these AEs was on a decline during the first 4 cycles of maintenance therapy (Figure 5).

In patients with advanced stage lung cancer, the goal of treatment is mainly to maintain or improve QoL. LCSS is used to evaluate 6 major symptoms associated with lung malignancies and their effect on overall symptom burden, functional activities, and overall QoL. In the CHMF group, after each of the 3 cycles of chemotherapy, median decrease in score from baseline was seen for fatigue (HR = 0.28, 0.07–0.88, *P* = 0.03) and interference with daily activities (HR = 0.40, 0.11–0.93, *P* = 0.04), indicating improvement. Improvement was also seen in the dyspnea score (HR = 0.38, 0.11–0.87, *P* = 0.03) after 2 cycles of chemotherapy. This finding suggests that the addition of CHMF to maintenance chemotherapy in the maintenance phase may have the effects of relieving symptoms and improving or maintaining QoL. No change or worsening in the LCSS scores was seen for all of the other symptoms, including overall symptoms, pain, and overall QoL.

KPS is an established prognostic factor for patient with advanced NSCLC. In our study, the KPS scores “improvement and stability rates” were 93.3% in the CHMF group vs. 70% in the placebo group (*P* = 0.047, 95% CI 0.03–0.935). With increase and stability of KPS scores, the number of chemotherapy cycles increased correspondingly, the total number of cycles being 239 in the CHMF group, compared with 122 in the placebo group. It is in line with the study of Shiguang Li and his colleagues. They demonstrated that CHM combined with first line chemotherapy also significantly increase one-year survival rate, immediate tumor response and improved KPS. Combined therapy remarkably reduced nausea and vomiting and prevented decrease of hemoglobin and platelet (Li et al., 2013).

TCM is characterized by holism and treatment based on syndrome differentiation. Briefly, it guides to maintain balance of the human body by regulating Yin and Yang, Qi and blood, as well as deficiency and excess. To deduce stratification factors, according to the inclusion criteria in this study, syndromes were limited to Qi deficiency and Yin deficiency. The therapeutic principles of lung cancer in TCM are strengthening healthy qi and eliminating pathogenic factors.Tonifying therapy is mainly used to alleviate the side effects induced by chemotherapy, for example, dry mouse and constipation, which are considered two of the typical symptoms of Qi and Yin deficiency, and the symptoms showed substantial improvement in this study. The following herbs we used in Yiqi formula have the function of tonifying qi: Milkvetch Root Radix Astragali, Codonopsis Radix, Atractylodis Macrocephalae Rhizoma and Indian Bread Poria. And the following herbs included in Yangyin formula have the function of nourishing yin: Coastal Glehnia Root, Fourleaf Ladybell Root, Cochinchinese Asparagus Root and Dwarf Lilyturf Tuber. The basic herbs were formulated to eliminate the pathogenic factors and demonstrated in many studies to have anti-cancer effect *in vivo* and *in vitro* (Feng et al., 2016; Wu et al., 2016, 2018). On one hand, tonifying herbal medicine enhances immunity by increasing the number of T-lymphocyte subtypes and natural killer cells, so as to improve the ability of the human body to fight against tumor cells (Zhuang et al., 2012). On the other hand, anti-cancer herbal medicine inhibits the growth of cancer. The combination of the two is commonly used in the treatment of NSCLC (Liu et al., 2008; Kou et al., 2017). During MT, keeping a favorable living state is the premise, while TCM can improve QoL and keep the body in balance. So, the treatment goal of TCM is similar to that of MT. We believe that TCM plus MT is more feasible (Xu et al., 2014).

We are also aware of some limitations in our study. First, since the sample size of our study was not large enough, we did not use stratified analysis for chemotherapy regimens and pathological types while analyzing the relationship between PFS and therapy model. The second limitation of this analysis is the consistently poor compliance with QoL assessment in the placebo group during maintenance therapy. The number of patients completing the questionnaire seems to reflect lower questionnaire distribution rate rather than a reduced likelihood of patients in that group completing the questionnaire. Third, because of complexity in composition, further basic research on the CHMF granule preparations is needed.

In conclusion, the results from the present study suggest that maintenance chemotherapy combined with CHMF may prolong PFS by relieving symptoms, improving QoL and alleviating the side effects. More attention should be paid to the role of Chinese herbal medications in combination therapy for advanced NSCLC.

## Availability of data and materials

To support the results reported, data and materials can be found on the website https://www.clinicaltrials.gov/ct2/show/NCT02900742?term=02900742&rank=1.

## Author contributions

LX, YaG, and LJ conceived and designed the experiments. LB, DZ, JY, JL, ZC, YJ, ZZ, WS, WZ, JX, and YaG performed the experiments and QW and SW wrote the paper. QW, SW, and PC collected and analyzed the data. LJ and PC provided technical expertise. YaG and LX provide assistance with revising this manuscript. All authors read and approved the manuscript.

### Conflict of interest statement

The authors declare that the research was conducted in the absence of any commercial or financial relationships that could be construed as a potential conflict of interest.
